# Linical efficacy of percutaneous endoscopic lumbar discectomy for the treatment of lumbar spinal stenosis in elderly patients: a retrospective study

**DOI:** 10.1186/s13018-020-01968-0

**Published:** 2020-09-24

**Authors:** Hua Li, Yufu Ou, Furong Xie, Weiguo Liang, Gang Tian, Hongyu Li

**Affiliations:** 1 Department of Spine Surgery, Guangxi Orthopedics and Traumatology Hospital, Nanning, China; 2 Department of Orthopaedics, People’s Hospital of Guangxi Zhuang Autonomous Region, Nanning, China

**Keywords:** Lumbar spinal stenosis (LSS), Elderly, Percutaneous endoscopic lumbar discectomy (PELD), Transforaminal approach, Interlaminar approach

## Abstract

**Background:**

Although percutaneous endoscopic lumbar discectomy (PELD) is increasingly being used to treat lumbar degenerative disease, the treatment of elderly patients with lumbar spinal stenosis (LSS) involves considerable uncertainty. The purpose of this study was to evaluate the safety and effectiveness of PELD for the treatment of LSS in elderly patients aged 65 years or older.

**Methods:**

In this retrospective review, 136 patients aged 65 years or older who underwent PELD to treat LSS were evaluated. The patients were divided into two groups, group A (ages 65–74) and group B (age ≥ 75), and perioperative data were analyzed. The Japanese Orthopaedic Association (JOA) score, visual analog scale (VAS) score, and MacNab classification were used to evaluate postoperative clinical efficacy.

**Results:**

All patients successfully underwent the operation with satisfactory treatment outcomes. Compared to preoperative scores, the self-reported scores or pain while performing daily activities were significantly improved in both treatment groups (*P* < 0.05). No statistically significant between-group differences were observed in operation time, intraoperative blood loss, postoperative bed rest, and postoperative hospital stay (*P* > 0.05). The overall postoperative complication rate was similar between the two groups. Moreover, no statistically significant differences in VAS-back pain scores, VAS-leg pain scores, JOA scores, and MacNab classification were found between the groups at the 3-month and 1.5-year follow-up examinations (*P* > 0.05).

**Conclusion:**

PELD is safe and effective for the treatment of LSS in elderly patients. Age is not a contraindication for decompressive lumbar spine surgery. PELD has advantages such as reduced trauma, fewer anesthesia-related complications, and a fast postoperative recovery. Elderly patients should be considered good candidates for lumbar decompression surgery using minimally invasive techniques.

## Introduction

Lumbar spinal stenosis (LSS) is a common condition among elderly individuals that severely impacts the quality of life [1]. Although conservative interventions have been performed for patients with severe LSS, surgical decompression is associated with a more favorable outcome compared to nonsurgical management [2–4]. Elderly patients are often contraindicated for surgical treatment due to their age, underlying disease, and the shortcomings of spinal decompression and fusion, such as significant trauma, high postoperative complication rate, and slow recovery [5, 6]. However, with the aging of society in China, the better understanding of LSS, and the higher demand for minimally invasive surgery, both doctors and patients prefer to treat LSS with a minimally invasive approach. In recent years, spinal minimally invasive technology has been advancing rapidly. Percutaneous full endoscopy (PE) has become the most minimally invasive method for the treatment of lumbar degenerative diseases. Its efficacy is similar to that of classic microendoscopic discectomy (MED), with less trauma and faster recovery. Moreover, PE can be completed under local anesthesia. With the advent of the “precise decompression” concept and continuous improvement of optical systems and endoscopic devices, the indications and intended uses of percutaneous endoscopic lumbar discectomy (PELD) are being expanded [7–10]. Few studies have been conducted in China or abroad to investigate the effect of surgical treatment in LSS patients aged 65 or over. In this study, we analyzed the clinical data of LSS patients aged 65 years or older to investigate the safety and clinical efficacy of PELD in elderly LSS patients.

## Materials and methods

### Inclusion/exclusion criteria

Inclusion criteria are as follows: (1) age ≥ 65 years, (2) a clinical syndrome predominantly characterized by unilateral lumbar radicular symptoms without severe back pain, (3) concordant computed tomography (CT) and magnetic resonance imaging (MRI) findings with clinical symptoms, (4) ineffective conservative treatment for 3 months or more or recurrent symptoms, and (5) complete data, with at least 1 year of follow-up.

Exclusion criteria are as follows: (1) back/lower-extremity pain due to injury, a tumor, tuberculosis, or severe osteoporosis; (2) lumbar instability on dynamic radiographs: translation greater than 3 mm or changes in angulation greater than 10° at one motion segment with lateral flexion and extension; (3) multisegment LSS with severe lumbar degenerative scoliosis or developmental deformity; (4) mental illness or an inability to cooperate; and (5) a history of previous lumbar surgery.

### General information

A total of 136 patients with single-segment LSS treated at our hospital between July 2015 and January 2017 met the entry criteria and were included in this study. To investigate the age-related effects on the results of spinal surgery, patients were divided into two groups. Group A included 35 males and 38 females, whose ages were between 65 and 74 years at the time of surgery, the average age at the time of surgery was 68.7 ± 2.3 years. While group B included 23 males and 40 females, whose ages were at least 75 years at the time of surgery, and the average age at the time of surgery was 80.1 ± 4.6 years. No significant difference in general information was observed between groups (*P* > 0.05, Table 1). All patients underwent lumbar X-ray (anteroposterior and lateral, and dynamic), MRI, and CT before the operation, as well as lumbar MRI or CT after the operation and during follow-up.

**Table 1:** Demographic features of 136 patients

Items	Group A (65–74 years)	Group B (≥ 75 years)	*P* value
Number of patients	73	63	
Age (years)	68.8 (65–74)	80.1 (75–96)	
Sex (M/F)	35/38	23/40	> 0.05
Comorbidities (*n*)			> 0.05
One	17	14	
Two	7	8	
Three or more	5	6	
Surgical level			>0.05
L2/3	3	1	
L3/4	7	4	
L4/5	37	40	
L5/S1	26	18	

### Surgical approaches

#### Preoperative preparation

Patients with medical conditions such as hypertension and diabetes had to have their condition under control before the operation.

#### PE system

Joimax PE system (Germany) and Ellman cryoablation and radiofrequency ablation system (USA) are used.

#### Transforaminal approach

**Fig. 1:**
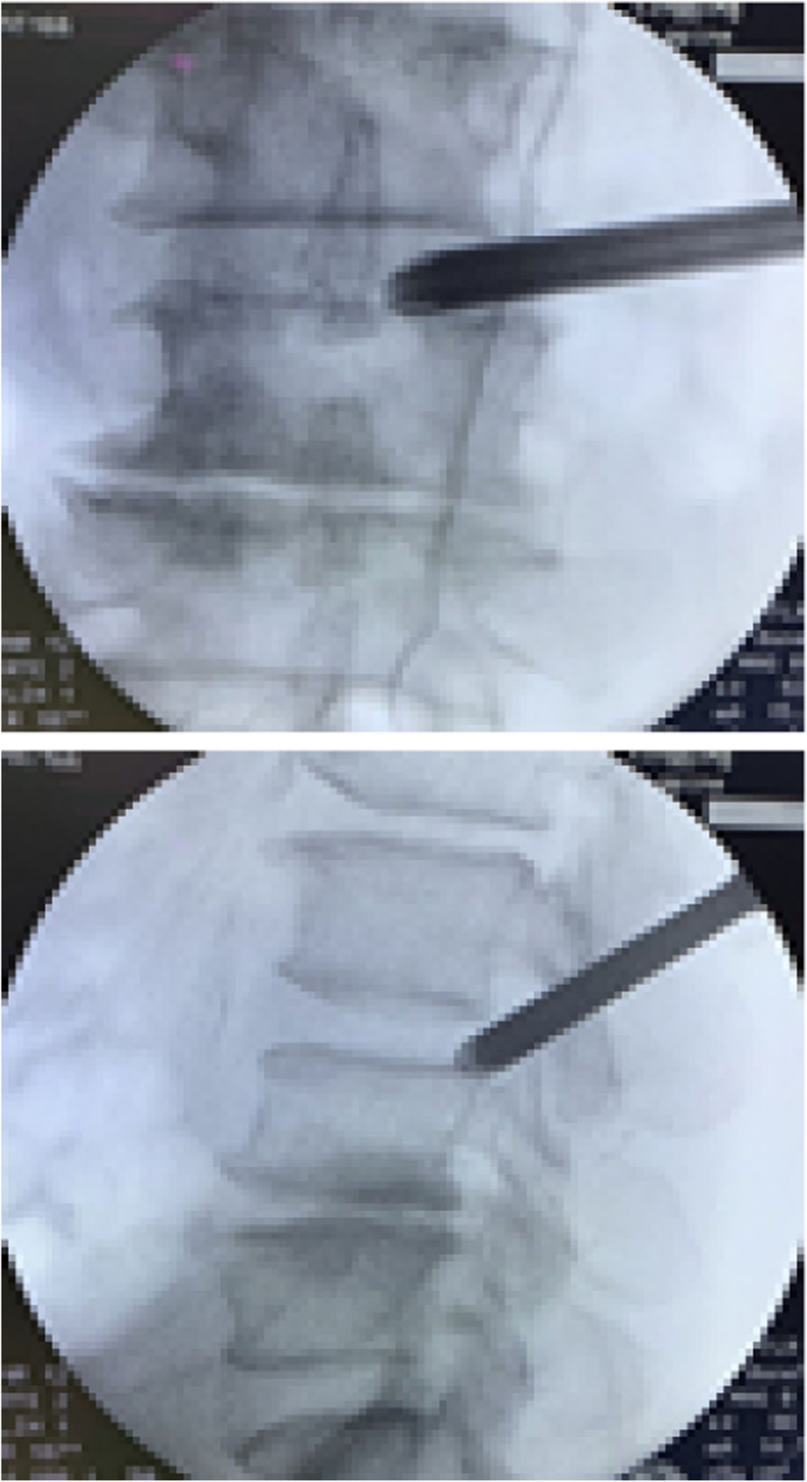
Transforaminal approach

The patient flexed his/her hips and knees and was placed in a lateral position (on the unaffected side). The patients received local anesthesia, and the puncture sites for the transforaminal approach were marked under the guidance of fluoroscopy. A needle was inserted and advanced to the tip of the superior facet of the vertebra inferior to the affected segment. A solution of 1% lidocaine and 0.25% ropivacaine was injected from the skin to the surrounding tissue of the facet for local anesthesia. An 18-gauge needle was inserted along the predefined channel and advanced to the anterior inferior edge of the superior facet of the vertebra inferior to the affected segment. A series of dilating catheters were sequentially used to dilate the surgical channel. The bone structure at the tip and ventral part of the superior facet was sawed or drilled until the circular saw or bone drill had reached the outer boundary of the spinal canal (Fig. 1) (this step was critical in elderly patients due to more severe lumbar vertebra degeneration, facet hyperplasia, and intervertebral foraminal stenosis). Next, a working catheter was placed via the intervertebral foramen and into the spinal canal; under endoscopic observation, various nucleus pulposus forceps and scissors with different angles were used to remove intervertebral disc tissues, hypertrophic ligamentum flavum, and some calcified tissues that were compressing the nerve. A grinding drill was used to polish osteoproliferative or calcified structures. During the operation, a radiofrequency bipolar device was inserted via the working channel to stop the bleeding, ablate the nucleus pulposus, loosen the nerve root, and then shrink the fibrous annular fissure in the working area.

#### Interlaminar approach

**Fig. 2:**
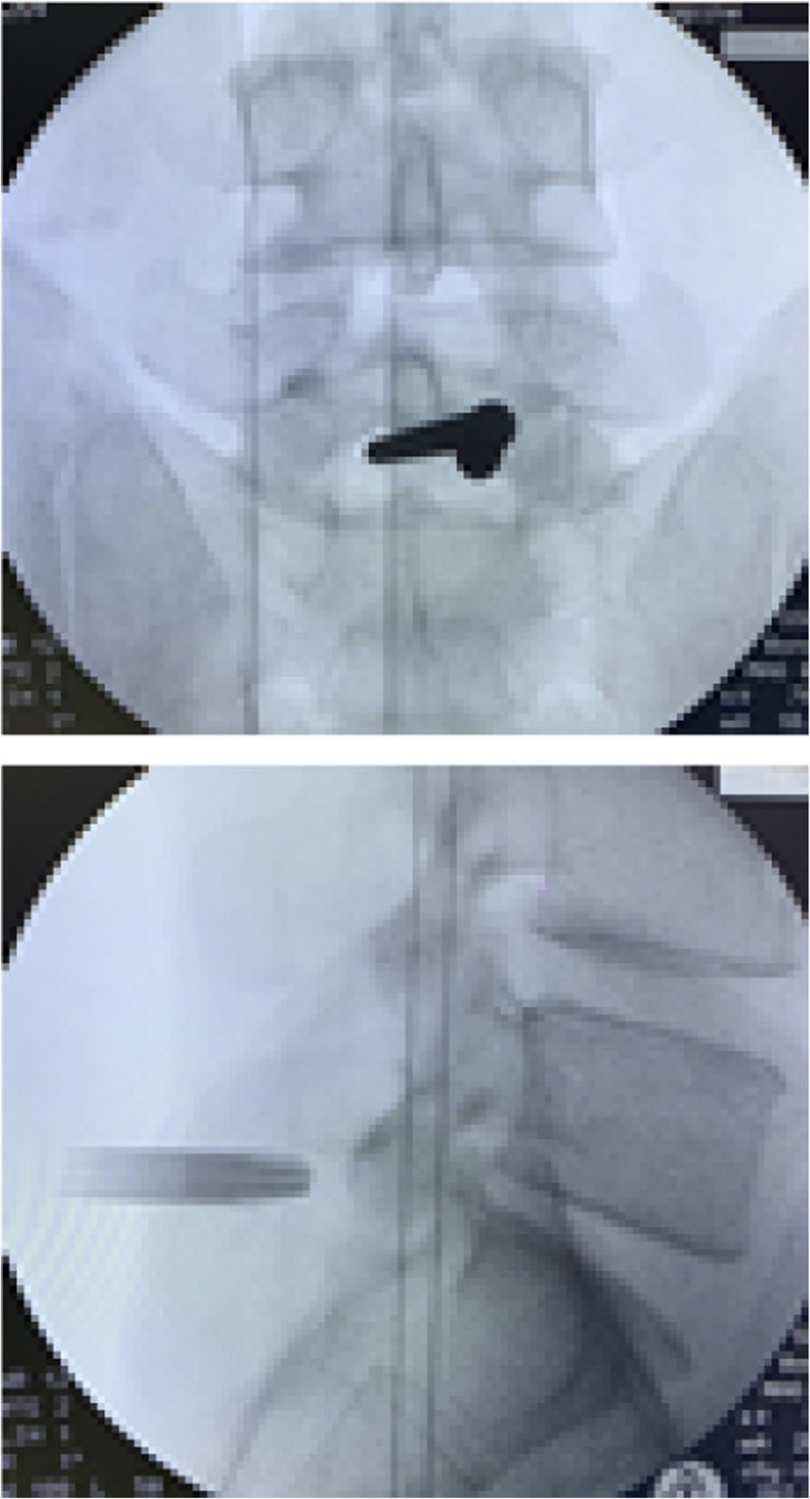
Interlaminar approach

The patient received general anesthesia and was placed in a prone position on the operating table. The laminar space of the affected segment was identified under the guidance of fluoroscopy. An 18-gauge needle was inserted into the skin approximately 0.5 cm next to the spinous process line of the affected side and advanced to the ligamentum flavum at the outer edge of the laminar space (Fig. 2). A dilator was then inserted by rotation. Under endoscopic observation, various nucleus pulposus forceps and scissors with different angles were used to remove the ligamentum flavum and lateral soft tissue. A grinding drill was used to polish osteoproliferative structures, including the ossified ligamentum flavum and hyperplasic facet; the tongue of the working cannula was inserted and rotated into the spinal canal to expose the spinal canal contents. The dural sac was revealed after the removal of some of the transparent adipose tissue. Next, the endoscopic channel and the outer working sheath were adjusted outward, upward, and downward by leverage to explore the location of the nerve root; and the working sheath was continuously adjusted to expose the nerve root and push it medially to protect it. After the protruding nucleus pulposus was removed, the spinal canal was carefully examined to remove any free nucleus pulposus materials. During the operation, a radiofrequency bipolar device was inserted via the working channel to stop bleeding, ablate the nucleus pulposus, explore the spinal canal decompression, thoroughly loosen the neural tube, and then shrink the fibrous annular fissure in the working area.

### Evaluation method

#### The following perioperative data were evaluated in both groups: operation time (min), intraoperative blood loss (ml), postoperative bed rest (h), postoperative hospital stay (d), and postoperative complications (n)

##### Postoperative observation and functional evaluation

The patients were followed-up at 1 week, 3 months, and 1.5 years postoperatively. The visual analog scale (VAS) and the Japanese Orthopaedic Association (JOA) score were used to evaluate the intensity of pain and lumbar spine function. Modified MacNab classification was used to evaluate clinical efficacy, as “excellent” (complete resolution of symptoms, ability to resume normal work and daily activities), “good” (minor symptoms, mild restriction in activities with no significant impact on work and daily activities), “fair” (improvement in symptoms, restrictions in activities with significant impact on normal work and daily activities), or “poor” (no improvement or worsening symptoms after treatment).

### Statistical analysis

Statistical analyses were performed using the SPSS 22.0 soft-ware (SPSS, Inc., Chicago, IL). Quantitative data were shown as the mean ± standard deviation, and qualitative data were expressed as the frequency (%). The preoperative and postoperative (1 week, 3 months, and 1.5 year) VAS scores of low back pain and leg pain and JOA scores were analyzed with repeated measures MANOVA. *P* < 0.05 was considered statistically significant.

## Results

All the patients were followed-up for 18 to 24 months (group A: 19.2 ± 3.2 months; group B: 19.6 ± 3.0 months).

### Evaluation of perioperative data

No statistically significant between-group differences were observed in operation time, intraoperative blood loss, postoperative bed rest, and postoperative hospital stay (*P* > 0.05). The overall postoperative complication rate was similar between the two groups (*P* > 0.05) (Table 2).

**Table 2:** General clinical results in the group A and group B

Items	Group A (65–74 years)	Group B (≥ 75 years)	*P* value
Operative time (min)	78.42 ± 20.34	81.98 ± 21.86	> 0.05
Blood loss (ml)	41.51 ± 10.06	45.79 ± 10.60	> 0.05
Postop. bed rest (h)	4.9 ± 1.82	5.2 ± 1.73	> 0.05
Postop. hospital stay (days)	2.7 ± 0.96	3.0 ± 0.87	> 0.05
Postop. complications and other events (*n*)	1	2	> 0.05

### Evaluation of postoperative functional measures

**Fig. 3:**
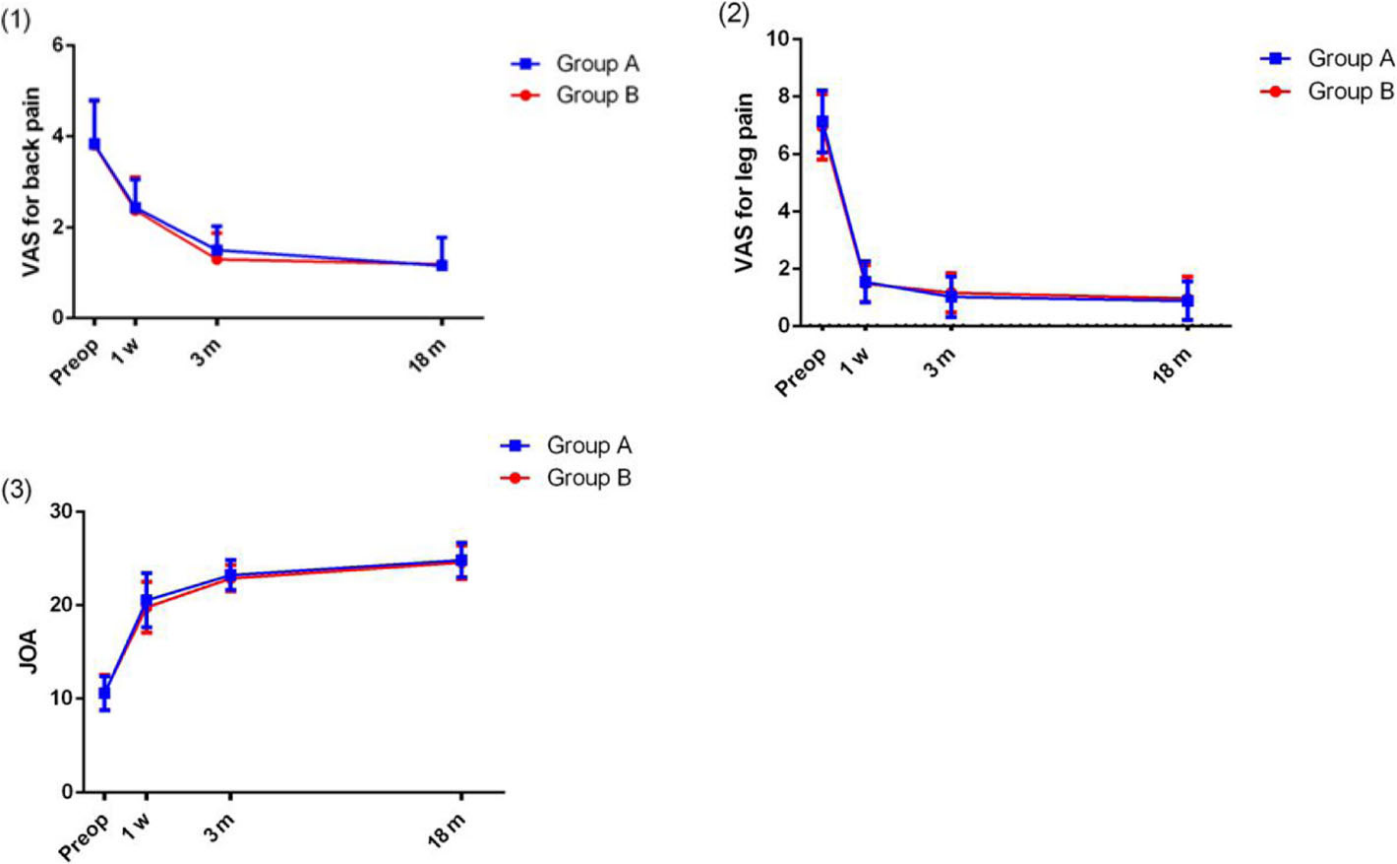
Clinical outcomes before and after endoscopic decompression at different follow-up time points in group A and group B. (1) Visual analog scale (VAS) scores for back pain. (2) VAS scores for leg pain. (3) Japanese Orthopaedic Association (JOA) scores

**Fig. 4:**
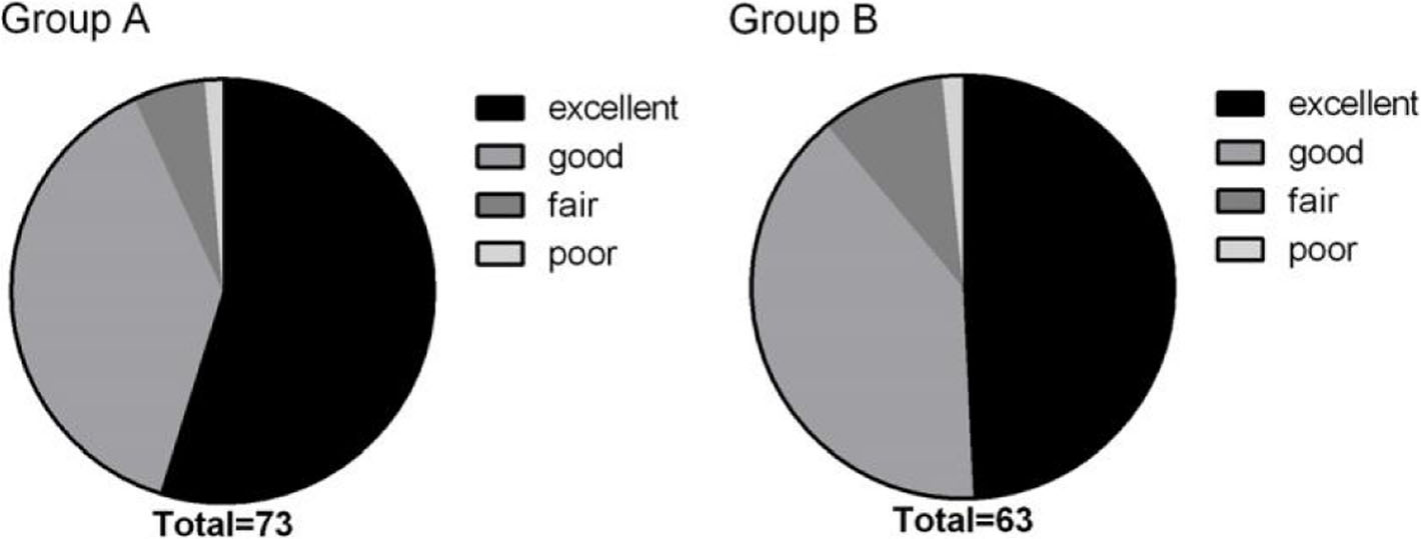
Satisfaction rates according to the modified MacNab criteria in group A and group B at the final review (18 months) postsurgery

**Fig. 5:**
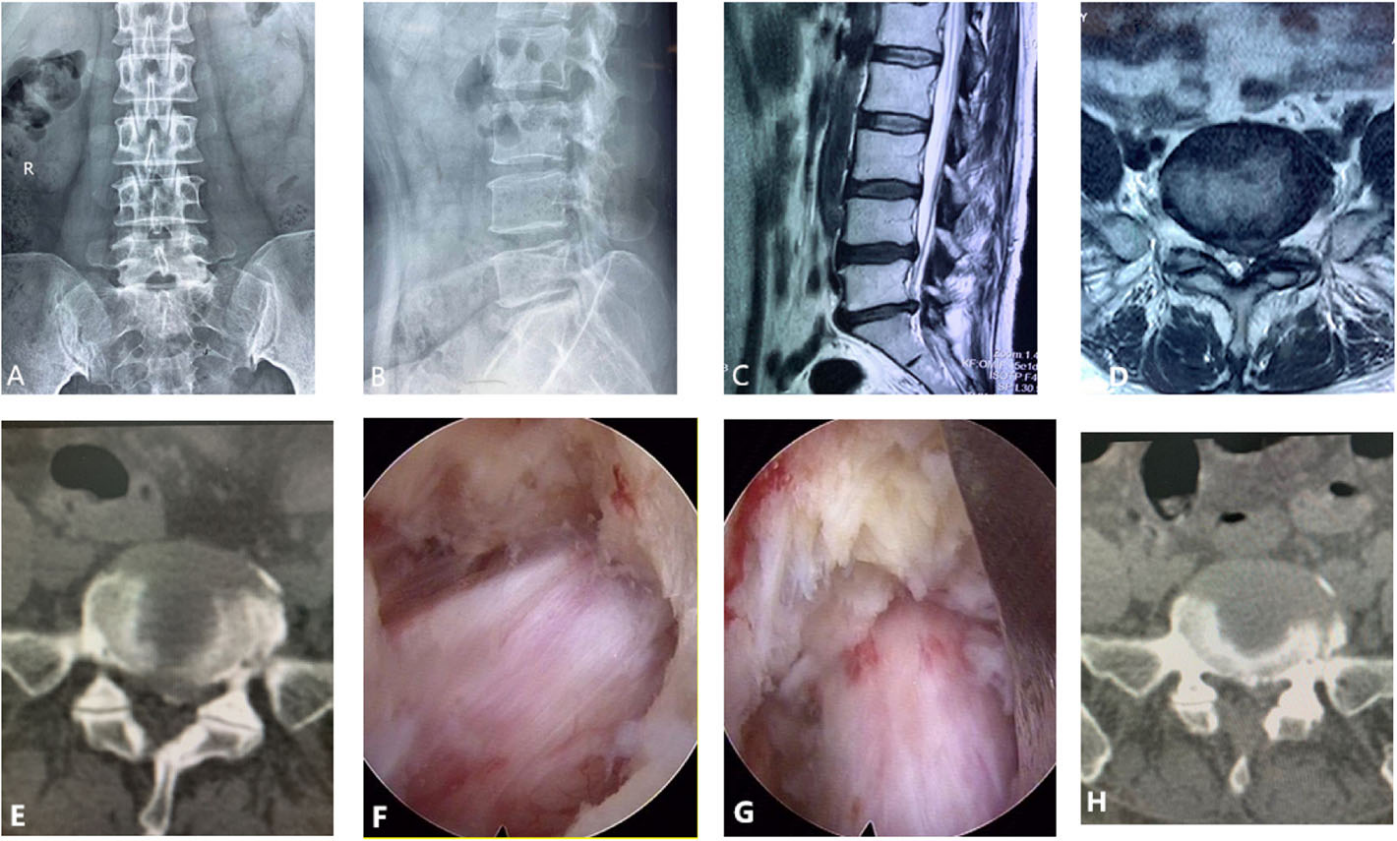
A female patient with lumbar spinal stenosis (LSS) who received PELD. **a**–**e** Preoperative X-ray radiography and computed tomography (CT) and magnetic resonance images (MRI) showing severe lateral recess stenosis and hypertrophy of the articular process and lumbar disc herniation with LSS at the left L4-5 level. **f**, **g** Intra-operative endoscopic images showing the medial side of the facet and osteophytes have been removed, and the neural tube has been completely decompressed. **h** Postoperative CT images showing the dorsal partial hypertrophied facet joint and ventral extruded disc had been removed

All patients successfully underwent the operation with satisfactory treatment outcomes. As shown in the data, the VAS scores of low back pain and leg pain, and JOA scores were significantly lower in all time-points at post-operation, when compared to those at pre-operation (*P* < 0.05) (Fig. 3). The trends of VAS scores of low back pain and leg pain, and JOA scores in group B were similar to those in group A (Fig. 3). No significant differences in the mean VAS score for back pain, the mean VAS score for leg pain, and the mean JOA score were found in all time-points at post-operation. Clinical outcomes according to the modified MacNab criteria are shown in Fig. 3. The good-to-excellent rate was 93.2% in group A and 89.9% in group B at the final review (Fig. 4). A thorough nerve root decompression, by removing the dorsal partial hypertrophied facet joint and ligament flavum and ventral extruded disc, was displayed on intra-operative endoscopic images or cross-sectional CT films (Fig. 5).

## Discussion

With social and economic development, societal aging has become an irreversible global trend in the twenty-first century. In 2016, the average life expectancy reached 76 in China [11]. As a result, the prevalence of degenerative LSS is increasing. However, chronic pain and discomfort from LSS may significantly impair psychosocial function and induce sleep disorders, depressive symptoms, and increased utilization of health care [12]. In recent years, PE has become the most minimally invasive treatment for lumbar degenerative disease, its indications are constantly being expanded, and it has proven efficacy for LSS [10, 13]. For elderly patients with LSS, while traditional surgical treatment can achieve extensive decompression and pain relief, it is associated with high risk and long recovery before resuming normal activities, and it takes longer to improve the quality of life. Elderly patients often have severe and complex underlying diseases, which exacerbate surgical risks and problems such as reduced spinal stability, prolonged bed rest, high complication rate, and high costs [6].

In this study, analysis of early efficacy showed that PE had the following advantages in elderly patients with LSS: (1) more extensive anesthesia indications and low risk from general or local anesthesia, low intraoperative blood loss, short operation time, and minimal intraoperative fluctuations in blood circulation; (2) minimal damage to the bone structure (only part of the thickened ligamentum flavum, protruding intervertebral disc tissue, and osteophytes on the medial side of the facet were removed during operation) and minimal impact on the stability of the posterior spine; (3) short postoperative bed rest and low complication rate (no postoperative infection or deep venous thrombosis was observed in this study); (4) effective relief of back/lower-extremity pain, with significant improvement in VAS and JOA scores on postoperative day 1 and at the last follow-up visit; (5) a short hospital stay, satisfactory improvement in symptoms after the operation, demonstrated efficacy, and low cost; and (6) age is not a contraindication for decompressive lumbar spine surgery.

## Physiological characteristics and surgical options for elderly patients with LSS

Elderly patients with LSS often have the following physiological and clinical characteristics: (1) LSS is characterized by degenerative changes such as facet joint hyperplasia and cohesion and ligamentum flavum hypertrophy due to long-term back pain–associated adverse lumbar stress and compensatory lumbar hyperplasia, as well as slow onset and prolonged disease course. Multiple segments are involved in most cases, and lumbar spondylolisthesis and scoliosis are observed in some cases. However, the symptoms of nerve injury are often inconsistent with imaging findings, and the location of affected segments is not clear. (2) Elderly patients have varying degrees of a severe and complex underlying disease, which limit treatment options and significantly increase the surgical risk and postoperative complication rate. (3) Elderly patients often have general organ insufficiency, requiring more complex perioperative management. Moreover, they are prone to incision infection or delayed healing, pulmonary infection, urinary infection, and gastrointestinal disorders after anesthesia and operation due to poor nutrition status and low immunity, which severely affect the postoperative efficacy. Therefore, for elderly patients with LSS, the goal of surgical treatment is to decompress completely while maintaining spinal stability. Moreover, depending on the patient’s physical condition, it is important to reduce anesthesia and operation time and minimize surgical trauma. While traditional surgical treatment can achieve extensive decompression and pain relief, it takes a long time for patients to resume normal activities and to improve the quality of life. Elderly patients often have severe and complex underlying diseases and are thus at increased risk for open surgery, reduced spinal stability, prolonged bed rest, high complication rate, and high costs [5, 6]. With the continuous advancement and development of PE, many researchers in China and abroad believe that PE is a safe and minimally invasive spinal technology with low blood loss, minimal postoperative scars, minimal nerve adhesions, minimal impact on the stability of the posterior spine, fast postoperative recovery, and the option of local anesthesia [8, 9, 14]. Therefore, PE can achieve the goal of “targeted and precise” decompression, while maintaining spine stability and minimizing surgical trauma. This study showed that PE achieved satisfactory outcomes with fast postoperative recovery and low complication rate in elderly patients.

### Is postdecompression fusion always required during the surgical treatment of lumbar spinal stenosis?

Researchers still debate the need for spinal fusion after decompression in LSS patients. In some cases, LSS is accompanied by degenerative spondylolisthesis. Many spine surgeons regard this as an absolute indication of fusion. Spinal surgery may impose a high risk of degenerative spondylolisthesis, and many surgeons therefore believe that performing fusion after decompression is the best course of action [15–17]. However, no evidence is available to support any benefits of fusion in LSS patients without spondylolisthesis [18, 19]. Several prospective studies with ≥ 5-year follow-ups [19, 20] have demonstrated better clinical results in cases without spinal fusion because spinal fusion may promote degeneration of adjacent segments and lead to a higher revision rate. Forsth et al. [21] conducted a large retrospective analysis of 8785 eligible patients. Patients who were lost to follow-up and who were followed-up for less than 2 years were excluded from the analysis. A total of 5390 patients were included in the analysis. The analysis included patients with or without lumbar spondylolisthesis before the operation. Various measures were evaluated during postoperative follow-up. The 2-year follow-up showed no significant difference in various clinical measures between the decompression group and the fusion group, regardless of the presence of lumbar spondylolisthesis before the operation. Moreover, the incidence of reoperation due to recurrent spinal stenosis or spinal instability was similar in the two groups (decompression group: 7%, fusion group: 8.1%). Lad et al. [22] conducted a retrospective cohort analysis and found that the 90-day complication rate after initial admission was significantly higher in the decompression-plus-fusion group than in the decompression-alone group, with no significant between-group difference in the revision rate during the ≥ 5-year follow-up. In addition, decompression plus fusion prolonged hospital stay and was associated with higher blood loss and costs. Other studies reached different conclusions regarding the reoperation rate. Ghogawala et al. [23] conducted a randomized clinical trial to investigate the efficacy of lumbar decompression plus fusion versus lumbar decompression alone for the treatment of grade I lumbar degenerative spondylolisthesis with spinal stenosis and found no significant difference in the Oswestry Disability Index (ODI) at 2 years after the operation between the groups. However, blood loss, hospital stay, and hospitalization costs were significantly higher or longer in the decompression-plus-fusion group than in the decompression-alone group. The reoperation rate was 14% and 34%, respectively. Some researchers believe that the high reoperation rate in the decompression-alone group was due to the clinical decision about revision, as surgeons are more inclined to perform spinal fixation in the case of unsatisfactory decompression alone but are less likely to perform revision in the case of unsatisfactory decompression plus fusion. These data indicate that fusion is probably no longer the best treatment for LSS. Further research is needed to investigate whether internal fixation is appropriate or necessary for LSS.

### Surgical strategies for PE in elderly patients with LSS

PE has become the most minimally invasive treatment of lumbar degenerative diseases. However, precise decompression is critical due to its limited operating field. It is important to perform a comprehensive assessment of imaging studies and physical examination before the operation in order to develop an individualized treatment plan. We believe that for elderly patients, the lumbar spinal canal is often associated with hypertrophic ligamentum flavum, facet joint hyperplasia, and lateral recess stenosis. Moreover, the degree of spinal canal stenosis on imaging findings is often discordant with the severity of clinical conditions. Therefore, in this study, the transforaminal approach was used in elderly patients whose primary symptom was lower-extremity radiating pain due to disc herniation and lateral recess stenosis, and the interlaminar approach was used in patients with long-term intermittent limping with central LSS on imaging studies for thorough decompression. For patients with long-term mild to moderate intermittent limping and recent severe lower-extremity radiating pain due to lateral disc herniation, the transforaminal approach was used under local anesthesia with good clinical efficacy. The selection of the specific approach is also related to the location of the affected segment. L5/S1 is usually blocked by the high crest, which hinders the placement and adjustment of a transforaminal channel. Therefore, for patients with L5/S1 spinal canal and high crest, the interlaminar approach was used under general anesthesia. During decompression, some of the thickened ligamentum flavum and protruding intervertebral disc tissue should be removed, and the osteophytes and calcified tissues at the edge of the lamina and the medial side of the facet should be polished to achieve thorough decompression of the spinal canal and/or neural tube. Percutaneous endoscopic lumbar spinal decompression is a minimally invasive surgical technique that is continuously developing, and it has a steep learning curve. The requirements for endoscopic skills are higher for the treatment of elderly patients with LSS than for lumbar disc herniation.

This study has some limitations due to its design and follow-up time. This is a retrospective analysis of data collected from a database to evaluate the safety and effectiveness of PELD for the treatment of LSS in elderly patients aged 65 or over. Due to the special characteristics and the high threshold for surgical treatment of elderly patients, we only included patients with single-segment LSS and excluded patients with apparent lumbar instability or multisegment stenosis. In addition, the present study lacked a control group of patients who received either conservative management or open surgery. Moreover, the sample size was small, so it may not have covered all possible complications, such as perioperative cardiac events and death. In addition, the follow-up time was short, and the long-term efficacy of minimally invasive surgery for LSS was confirmed only in some patients with a longer follow-up time.

In summary, with the rapid development of minimally invasive technology, spinal endoscopic techniques enable individualized treatment plans for LSS. For elderly LSS patients with severe and complex underlying diseases, PELD is undoubtedly a better option, with recognized advantages such as a short operation time, a low surgical risk, minimal impact on spinal stability, a fast postoperative recovery, and proven efficacy. Patients who were previously considered ineligible for lumbar spinal surgery may be qualified for this treatment. Thus, more elderly patients will have an opportunity to undergo minimally invasive treatment for LSS and experience improved quality of life. Our results may help surgeons better understand and perform minimally invasive lumbar decompression in elderly patients.

## Data Availability

The datasets generated and analyzed during the current study are available from the corresponding author upon reasonable request.

## Abbreviations

LSS: Lumbar spinal stenosis
PE: Percutaneous full-endoscopy
MED: Microendoscopic discectomy
MRI: Magnetic resonance imaging
CT: Computed tomography
PELD: Percutaneous endoscopic lumbar discectomy
